# The Pyroptosis-Related Signature Predicts Diagnosis and Indicates Immune Characteristic in Major Depressive Disorder

**DOI:** 10.3389/fphar.2022.848939

**Published:** 2022-05-19

**Authors:** Zhifang Deng, Jue Liu, Shen He, Wenqi Gao

**Affiliations:** ^1^ Department of Pharmacy, The Central Hospital of Wuhan, Tongji Medical College, Huazhong University of Science and Technology, Wuhan, China; ^2^ Division of Mood Disorders, Shanghai Mental Health Center, Shanghai Jiao Tong University School of Medicine, Shanghai, China; ^3^ Institute of Maternal and Child Health, Wuhan Children’s Hospital (Wuhan Maternal and Child Healthcare Hospital), Tongji Medical College, Huazhong University and Technology, Wuhan, China

**Keywords:** pyroptosis, diagnostic, MDD, immune, gene cluster analysis

## Abstract

Pyroptosis is recently identified as an inflammatory form of programmed cell death. However, the roles of pyroptosis-related genes (PS genes) in major depressive disorder (MDD) remain unclear. This study developed a novel diagnostic model for MDD based on PS genes and explored the pathological mechanisms associated with pyroptosis. First, we obtained 23 PS genes that were differentially expressed between healthy controls and MDD cases from GSE98793 dataset. There were obvious variation in immune cell infiltration profiles and immune-related pathway enrichment between healthy controls and MDD cases. Then, a novel diagnostic model consisting of eight PS genes (*GPER1*, *GZMA, HMGB1*, *IL1RN*, *NLRC4*, *NLRP3*, *UTS2*, and *CAPN1*) for MDD was constructed by random forest (RF) and least absolute shrinkage and selection operator (LASSO) analyses. ROC analysis revealed that our model has good diagnostic performance, AUC = 0.795 (95% CI 0.721–0.868). Subsequently, the consensus clustering method based on 23 differentially expressed PS genes was constructed to divide all MDD cases into two distinct pyroptosis subtypes (cluster A and B) with different immune and biological characteristics. Principal component analysis (PCA) algorithm was performed to calculate the pyroptosis scores (“PS-scores”) for each sample to quantify the pyroptosis regulation subtypes. The MDD patients in cluster B had higher “PS-scores” than those in cluster A. Furthermore, we also found that MDD patients in cluster B showed lower expression levels of 11 interferon (IFN)-α isoforms. In conclusion, pyroptosis may play an important role in MDD and can provide new insights into the diagnosis and underlying mechanisms of MDD.

## Introduction

Pyroptosis, a programmed cell death mode closely related to the inflammatory response, plays an important role in a variety of physiological processes and disease progression (Broz et al., 2020). The characteristics of pyroptosis include activation of caspase-1, 4, 5, and 11; formation of cell membrane pores mediated by gasdermin protein; cell swelling and rapid rupture; and release of intracellular inflammatory factors (Shi et al., 2017). Therefore, inflammatory vesicles, gasdermin protein, and pro-inflammatory cytokines are key factors involved in pyroptosis. The expressions and functions of these core regulatory components influence pyroptosis progression. Further study of these regulatory components may help to clarify the role of pyroptosis in disease pathogenesis (Ahechu et al., 2018).

Major depressive disorder (MDD) is a serious neuropsychiatric disorder and a leading cause of suicide (Lépine and Briley, 2011). The incidence of depression is increasing annually to rank third among global disease burdens (Malhi and Mann, 2018). The pathogenesis of depression is complex, and inflammation is one of the main pathogenic factors (Troubat et al., 2021). Inflammation results from abnormal immune system activation. The imbalance of immune cells in the body can lead to illness, including mental illness such as MDD. Patients with depression show dysregulation of the innate and adaptive immune systems; for example, monocyte activation, decreased T-cell number and/or activity, and increased production of pro-inflammatory cytokines (Beurel et al., 2020). Excessive inflammation caused by pyroptosis and the release of various inflammatory factors after cell rupture may aggravate the disease development by forming an inflammatory immune microenvironment (Xia et al., 2019).

Comprehensive analysis of pyroptosis characteristics alteration in depression may be a key strategy for diagnosis and physiopathologic mechanism exploration of depression. Due to technical limitations, previous studies were limited to one or two key factors of pyroptosis. However, disease occurrence and progression involve a series of factors that form a highly synergistic network. Nowadays, the developments of high-throughput genomics technology and bioinformatics analysis have helped researchers to study genes expression profiles at the genomic level, generated new ideas for the interpretation of genomic results, and provided an ideal resource for the comprehensive analysis of pyroptosis and immune regulation in MDD (Gururajan et al., 2016; Ferrúa et al., 2019; Takahashi et al., 2019). In this study, we first established a novel diagnostic model by eight pyroptosis-related genes (PS genes) for MDD based on the GSE98793 dataset from the Gene Expression Omnibus (GEO) database. We found that MDD patients could obtain a good clinical benefit based on this model. Secondly, we explored the role of pyroptosis in physiopathologic mechanism of MDD. According to PS genes, data from patients with depression were clustered and two MDD subtypes were identified. We observed different immune properties and biological functions of these two subtypes. In all, our present study indicated that pyroptosis plays an important role in depression occurrence and progression, which may guide depression diagnosis, treatment and intervention plans.

## Materials and Methods

### Data Acquisition and Processing

GSE98793 dataset, the expression profile of whole blood samples, was downloaded from the Gene Expression Omnibus (GEO) database. This dataset totally included 128 MDD cases (64 with anxiety symptoms and 64 without) and 64 healthy controls. 64 MDD cases without anxiety symptoms and 64 healthy controls were included in our analysis. All the participants of GSE98793 were from the GlaxoSmithKline–High-Throughput Disease-specific target Identification Program (GSK-HiTDiP) study. The MDD patients were evaluated by the semi-structured Schedule for Clinical Assessment in Neuropsychiatry (SCAN) (Wing et al., 1990), which was administered by trained staff. Furthermore, patients had a diagnosis of recurrent MDD (at least two episodes of depression satisfying DSM-IV or ICD10 criteria) were included as well. The exclusion criteria were as follows: if they had experienced mood incongruent psychotic symptoms, a lifetime history of intravenous drug use or diagnosis of drug dependency, depression secondary to alcohol or substance abuse or depression as clear consequence of medical illnesses or use of medications. Patients with co-morbid anxiety disorders, with the exception of obsessive compulsive and post traumatic stress disorders, were included. Patients with diagnosis of schizophrenia, schizoaffective disorders and other axis I disorders were excluded from the study (Leday et al., 2018). The detail information of participants included in the present study was showed in Table 1. GPL570 (Affymetrix Human Genome U133 Plus 2.0 Array) was detection platforms for GSE98793. Gene symbols were used to annotate the downloaded gene probes, eliminate probes without matching, and retain any gene probes with multiple matching.

**TABLE 1:** Clinical and demographic characteristics of participants (GSE98793) included in the present study.

	All participants	MDD	Healthy controls
	128	64	64
Gender			
Male	32	16	16
Female	96	48	48
Comorbidities			
Anxiety	0	0	0
Without anxiety	128	64	64
Age (years)	52.06 ± 11.49	52.03 ± 11.41	52.09 ± 11.66

### Screening for Pyroptosis-Related Differentially Expressed Genes

“Limma” package (R Foundation for Statistical Computing) was used for gene differential expression analysis with the processed gene expression matrix (Diboun et al., 2006). Before the bioinformatic analysis, all the samples were tested in two batches, and batch information could be extracted from phenotypic data. Thus, removeBatchEffect from the limma package was used to remove the batch effect (Ritchie et al., 2015). We conducted the gene differential expression analysis with set threshold: |log_2_(FC)|>0.1 and Benjamini–Hochberg-adjusted *p* < 0.05. Finally, we identified 2,216 DEGs in GSE98793. A total of 184 PS genes were obtained by inputting the keyword “pyroptosis” in the GeneCards database. The overlap of 2,216 DEGs in GSE98793 dataset and 184 PS genes were defined as pyroptosis-related DEGs, totally 23 pyroptosis-related DEGs acquired.

### Screening MDD-Specific Genes and Constructing Diagnostic Model for MDD

We applied random forest (RF) and least absolute shrinkage and selection operator (LASSO) to establish a diagnostic model for MDD. First, we used RF to screen candidate MDD-specific genes from pyroptosis-related DEGs. RF is a general technique for the training and prediction of samples based on the classification tree. The number of decision trees (ntree) and the value of MTRY in this study were 300 and 4, respectively. The RF was performed by R package “randomforest” (Kursa, 2014). Subsequently, to reduce the number of genes in the model and to solve the multicollinearity problem in regression analysis, we used LASSO logistic regression to screen feature genes and then construct a diagnostic model for MDD. The “glmnet” package was applied for LASSO algorithm (Friedman et al., 2010). Finally, a receiver operating characteristic (ROC) curve was created to investigate whether the built model could effectively predict MDD.

### Internal and External Validation for Diagnostic Model

10-fold cross-validation as internal validation method was performed to confirm the predictive performance of our diagnostic model for MDD (Martinez et al., 2011). We chosed 10-fold cross-validation because 10-fold cross-validation techniques could test all data in the dataset and produce stable predictive accuracy. Therefore, the 10-fold cross-validation method with 2000 iterations of resampling was used for internal validatIon.

GSE76826 was used as an external validation dataset to examine the universality and reliability of the diagnostic model. GSE76826 dataset (expression profiling by array) included 20 MDD patients and 12 healthy controls (Miyata et al., 2016).

In addition to this, we examined the effectiveness of this diagnostic model in other mental illness as well. The GSE38484 dataset was used to analyze the diagnostic model. This dataset, based on the microarray platform of the Illumina HumanHT-12 V3.0 expression beadchip (GPL6947), included 96 healthy controls and 106 schizophrenia patients (Van Eijk et al., 2015).

The same model and the same coefficient were conducted for GSE76826 and GSE38484. The characteristic information of participants in GSE76826 and GSE38484 datasets were exhibited in Tables 2, 3.

**TABLE 2:** Clinical and demographic characteristics of participants (GSE76826).

Dataset		Participants	Age (years)	Source
GSE76826	Healthy	18	48.44 ± 10.82	PBMCs
	Male	10	47.30 ± 11.47	
	Female	8	49.88 ± 10.52	
	MDD	16	56.5 ± 9.96	
	Male	10	46.30 ± 10.18	
	Female	6	46.83 ± 10.53	

**TABLE 3:** Clinical and demographic characteristics of participants (GSE38484) included in the present study.

Dataset		Participants	Age (years)	Source
GSE38484	Healthy	96	39.46 ± 12.47	Whole blood
	Male	42	39.52 ± 14.41	
	Female	54	39.15 ± 14.15	
	SCZ	106	39.58 ± 10.74	
	Male	76	39.47 ± 10.50	
	Female	30	39.87 ± 11.49	

### Diagnostic Markers Verified by Postmortem Brain Tissue Samples

GSE53987 was based on the platform of the Affymetrix Human Genome U133 Plus 2.0 Array (Lanz et al., 2019). There were 17 subjects with MDD and 18 healthy controls in this dataset, and three brain regions (hippocampus, prefrontal cortex, and striatum) were included. We used the GSE53987 dataset to verify the diagnostic model. The characteristic information of participants in GSE53987 dataset were showed in Table 4.

**TABLE 4:** Clinical and demographic characteristics of participants (GSE53987) included in the present study.

Dataset		Participants	Age (years)	Source
GSE53987	Healthy	12	62.50 ± 9.39	Striatum
	Male	5	58.00 ± 6.44	
	Female	7	65.71 ± 10.24	
	MDD	20	71.45 ± 11.71	
	Male	9	68.56 ± 14.45	
	Female	11	73.82 ± 8.93	

### Consensus Clustering of 23 PS Genes by Partitioning Around Medoids

Consensus clustering is an algorithm used to identify subgroup members and verify subgroups based on resampling. We performed consensus clustering with PAM mehod (Wilkerson and Hayes, 2010) to identify distinct pyroptosis regulation clusters according to the expression profiles of 23 PS genes. PCA was then used to further validate different regulation clusters.

### Immune Cell Infiltration Estimation by ssGSEA

Single-sample gene set enrichment analysis (ssGSEA) was used to quantify the relative abundance of 28 immune cell types related to immune response. In ssGSEA, the relative abundance of each immune cell was expressed as an enrichment score that was normalized to a uniform distribution of 0–1. A deconvolution approach CIBERSORT (http://cibersort.stanford.edu/) was used to evaluate the relative abundances of 22 distinct leukocyte subsets with gene expression profiles in the blood samples.

### Gene Set Variation Analysis and Gene Ontology Annotation

We utilized GSVA analysis by “GSVA” package (Hänzelmann et al., 2013) to explore the differentiation in biological processes between different pyroptosis regulation clusters. The well-defined biological signatures were derived from the Hallmark geneset (MSigDB database v7.1) (Denny et al., 2018). The GO annotation for different clusters was performed using the R package “clusterProfiler” (Kursa, 2014) with a false discovery rate (FDR) cutoff of <0.01.

### Identification of DEGs in Distinct Pyroptosis Regulation Subtypes

The consensus clustering algorithm classified MDD patients into two distinct pyroptosis regulation subtypes. We next identified DEGs between two different clusters using the “limma” package. Specifically, gene expression data were normalized using “voom” function and then inputted to the “lmFit” and “eBayes” functions to calculate the differential expressed statistics. The selection criteria were an adjusted P of <0.01 and |FC| of >1.0.

### Construction of the Pyroptosis Score

To quantitatively analyze the pyroptosis subtypes, PCA was used to quantify the pyroptosis level of individual patients. First, PCA was used to distinguish pyroptosis subtypes. Then, the formula was performed to measure the pyroptosis scores (PS) as following: PS score = PC1_i_, where PC1 represents principal component 1 and i represents DEG expression.

## Results

### Study Design

The framework and workflow are described as following. First, the characteristic information and gene expression profiles of MDD cases and healthy controls in GSE98793, GSE76826, and GSE53987 were obtained from the GEO database (https://www.st-va.ncbi.nlm.nih.gov/gds/?term=). Using GSE98793 dataset, we developed a novel diagnostic model for MDD based on PS genes by machine learning methods (RF and LASSO). GSE76826 and GSE53987 datasets were used as external validation and postmortem brain tissue samples valiadtion. Furthermore, GSE38484 dataset (96 healthy controls and 106 schizophrenia patients) was used to examine whether this diagnostic model was unique for MDD. Then, immune cell infiltration profiles and immune-related pathway enrichment were compared between healthy controls and MDD cases as well.

To discover the connections between PS genes and MDD subtypes, MDD cases were divided into two subtypes (A and B clusters) by consensus clustering analysis according to the PS genes expression profiles. The particular immune and biological characteristics of these two clusters were observed.

We developed a pyroptosis-related signature score, the “PS-score,” to quantify the pyroptosis phenotype subtype.

### Identification of DEGs Between Healthy Controls and MDD Cases

**FIGURE 1:**
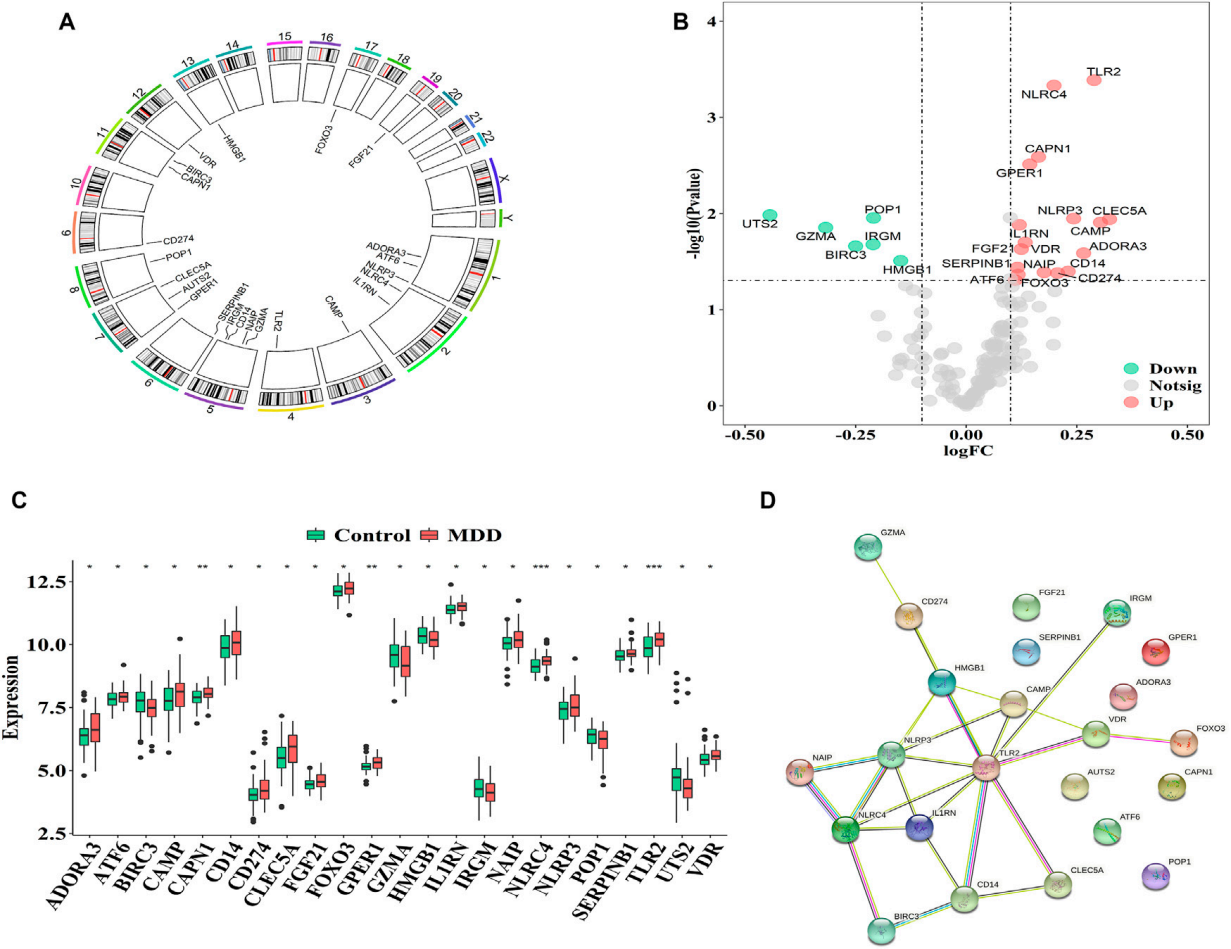
Characterization of pyroptosis-related genes in MDD **(A)**. The location of pyroptosis-related genes on chromosomes by GSE98793 **(B–C)**. The Volcano and heat map of 23 differentially expressed pyrotopsis-related genes **(D)**. Box plot of differential expression of 23 differentially expressed pyrotopsis-related genes in control and depression samples. Adjusted *p*-values were showed as: ns, not significant; *p < 0.05; **p < 0.01; ***p < 0.001.

The GSE98793 dataset and Genecard database included 2,216 DEGs and 184 PS genes, respectively, among which 23 PS genes with significant expression differences were distributed on chromosomes as shown in Figure 1A. The upregulated genes included C-type lectin member 5 A (*CLEC5A*); cathelicidin antimicrobial peptide (*CAMP*); Toll-like receptor 2 (*TLR2*); adenosine A3 receptor (*ADORA3*); NOD-, LRR-, and pyrin domain-containing protein 3 (*NLRP3*); cluster of differentiation 14 (*CD14*); *CD274*; NLR family CARD domain containing 4 (*NLRC4*), NLR family apoptosis inhibitory protein (*NAIP*); calpain 1 (CAPN1), G-protein coupled estrogen receptor 1 (*GPER1*); Vitamin D receptor (*VDR*); fibroblast growth factor 21 (*FGF21*); interleukin-1 receptor antagonist (*IL1RN*); forkhead box O3 (FOXO3); serpin family B member 1 (*SERPINB1*); and activating transcription factor 6 (*ATF6*). The downregulated genes included high mobility group box 1 (*HMGB1*), processing of precursor 1 (*POP1*)*,* immunity-related GTPase family (*IRGM*)*,* baculoviral IAP repeat-containing protein3 (*BIRC3*)*,* granzyme A (*GZMA*), and urotensin-II (*UTS2*) (Figures 1B,C).

Interactions among the 23 pyroptosis-related DEGs were observed, and each gene was mapped to the STRING database to show their interaction relationships (interaction minimum>0.9, highest confidence) and visualize in Cytoscape.

There were totally 54 interaction relationships among 23 pyroptosis-related DEGs. Figure 1D indicated the protein-protein interaction (PPI) network diagram.

**FIGURE 2:**
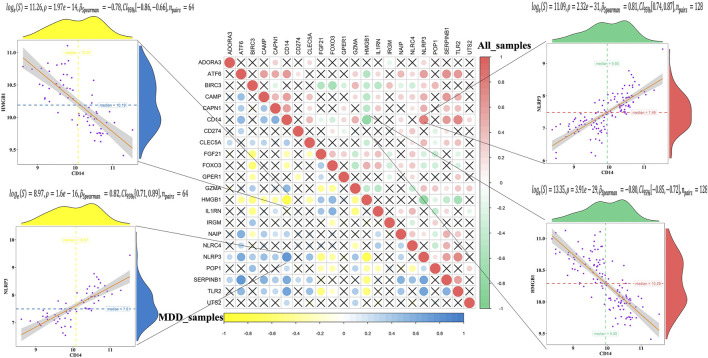
Correlation analysis of 23 differentially expressed pyroptosis-related genes in all samples and MDD samples.

The correlation analysis showed that CD14 was significantly positively correlated with NLRP3, while CD14 was significantly negatively correlated with HMGB1 in all healthy controls and MDD samples (Figure 2). These results indicated that expression imbalances of PS genes played important roles in the occurrence and development of MDD.

### A Diagnostic Model for MDD Constituting of PS Genes

**FIGURE 3:**
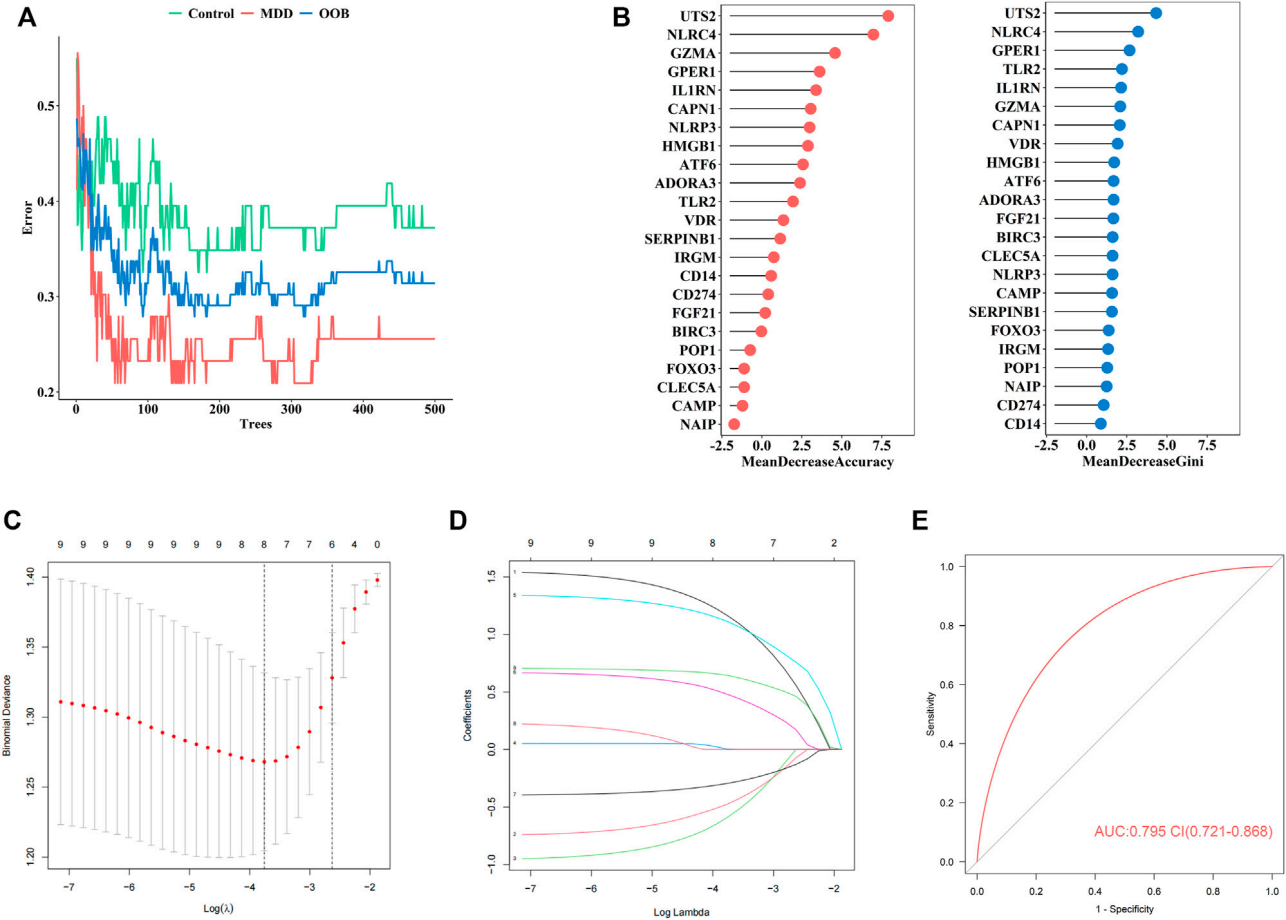
The construction of our diagnostic model for MDD **(A)**. Error between the number of decision trees and different groups **(B)**. The MeanDecreaseAccuracy and MeanDecreaseGini of 23 pyroptosis-related genes **(C)**. Screening of the optimal parameter (using lambda.1se as the best lambda) at which the vertical lines were drawn **(D)**. LASSO coefficient profiles of the 23 differentially expressed PS genes plus age and sex **(E)**. The ROC value of our diagnostic model in GSE98793 dataset.

Firstly, the RF was used to screen MDD-specific genes that optimally differentiated MDD cases from healthy controls. When the number of decision trees reached 300, the error changes of the three kinds gradually decrease (Figure 3A). The 23 PS genes identified by MeanDecreaseAccuracy and MeanDecreaseGini were showed in Figure 3B. The top 10 of these—*UTS2, NLRC4, GZMA, GPER1, IL1RN, CAPN1, NLRP3, HMGB1, ATF6*, and *ADORA3—*were candidate genes.

Secondly, to establish an MDD-specific diagnostic model, LASSO regression was conducted based on the above 10 genes and basic phenotype information (age and sex), we contained eight genes (*GPER1, GZMA, HMGB1, IL1RN, NLRC4, NLRP3, UTS2, CAPN1*) according to lambda.1se (Figures 3C,D).

Furthermore, to improve the diagnostic efficiency of biomarkers, a novel diagnostic risk score was constructed by multiplying the gene expression. The total risk score was imputed as follows: (1.163 × *GPER1* expression level) + (−0.474 × *GZMA* expression level) + (−0.588 × *HMGB1* expression level) + (0.003 × *IL1RN* expression level) + (1.111 × *NLRC4* expression level) + (0.479 × *NLRP3* expression level) + (−0.294 × *UTS2* expression level) + (0.644 × *CAPN1* expression level). Receiver operating characteristic (ROC) analysis was used to evaluate the diagnostic ability of eight genes, which showed a favorable diagnostic value, with an AUC of 0.795 (95% CI 0.721–0.868) (Figure 3E). The coefficient of eight genes were showed in Table 5.

**TABLE 5:** The coefficient of eight genes.

Gene	Coefficient
GPER1	1.162
GZMA	−0.474
HMGB1	−0.588
IL1RN	0.003
NLRC4	1.112
NLRP3	0.479
UTS2	−0.294
CAPN1	0.644

### Internal and External Validation of Diagnostic Model Performance

**FIGURE 4:**
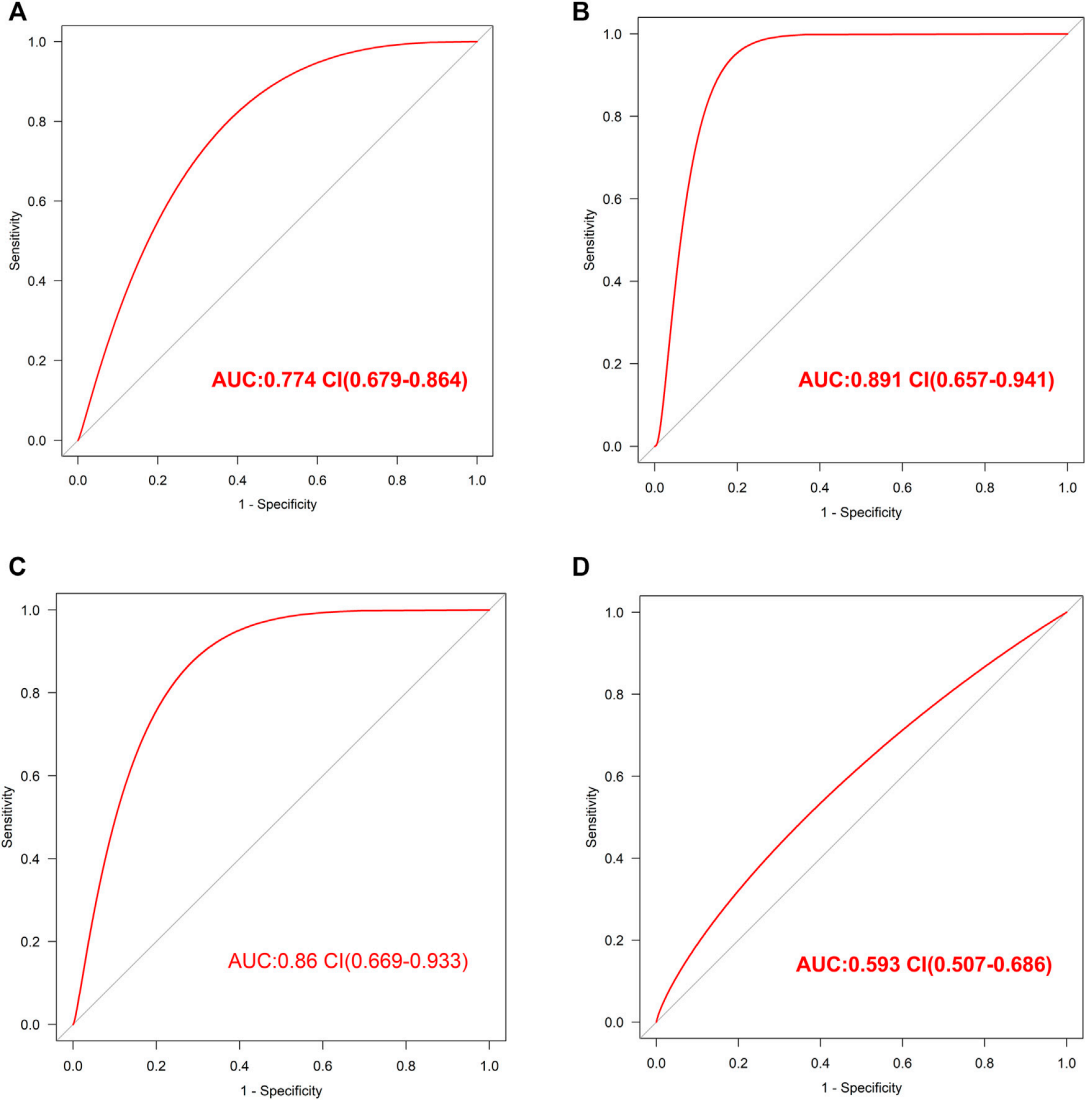
The validation of this diagnostic model **(A)**. The ROC value in internal validation of this diagnostic model **(B)**. The ROC value in external validation of this diagnostic model by GSE76826 dataset **(C)**. The ROC value in validation of this diagnostic model by GSE53987 dataset **(D)**. The ROC value in validation of this diagnostic model by GSE38484 dataset.

The internal validation of our diagnostic model was performed by 10-fold cross-validation (*n* = 2000). The model demonstrated good discrimination (bias-corrected AUC = 0.774, 95% CI = 0.679–0.864) (Figure 4A). The external analysis using independent dataset GSE76826 revealed a good performance of our diagnostic model for stratifying MDD patients (AUC = 0.891, 95% CI = 0.657–0.941) (Figure 4B).

Furthermore, the accuracy of our diagnostic model was validated using brain tissue GSE53987 dataset. The AUC was 0.86 (95% CI = 0.669–0.933), which also indicated good performance (Figure 4C).

In order to observe the effectiveness of this diagnostic model in other mental illness, the analysis of schizophrenia GSE38484 dataset was performed. The AUC of GSE38484 dataset was 0.593 (95% CI = 0.507–0.686) (Figure 4D), suggesting that this diagnostic model was not effective and accurate for schizophrenia. We speculated that our diagnostic model constituting of eight PS genes was more appropriate for MDD.

### Immune Cell Infiltration Profile and Immune-Related Pathway Enrichment Between Healthy Controls and MDD Cases

Many studies have indicated that MDD is accompanied by immune dysregulation, pyroptosis is closely related to immune response as well, thus we want to further explore the relationship between pyroptosis and immune response in MDD. The differences in immune cell infiltration profile and immune-related pathway enrichment between healthy controls and MDD cases were quantified.

**FIGURE 5:**
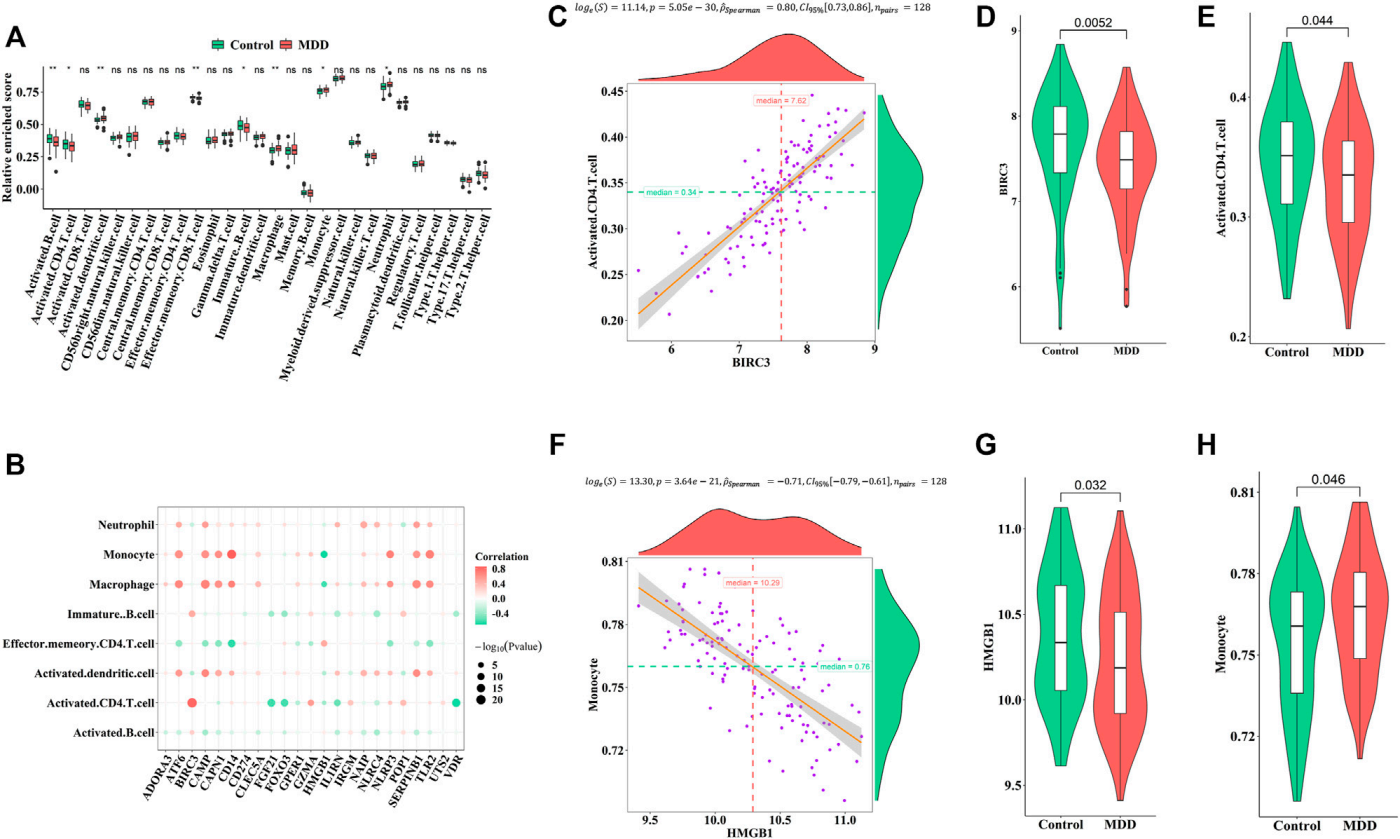
Correlation between pyroptosis-related genes and immune infiltrating cells **(A)**. Differences in the abundance of immune infiltrating cells between healthy control and MDD samples **(B)**. Correlation between pyroptosis-related genes and immune infiltrating cells **(C–E)**. The most positive correlation between immune infiltrating and pyroptosis-related genes **(F–H)**. The most negative correlation between immune infiltrating and pyroptosis-related genes. Adjusted *p*-values were showed as: ns, not significant; *p < 0.05; **p < 0.01.

Firstly, ssGSEA was used to calculate the relative abundance of immune cells in each sample. Eight of the immune cell types discovered significant changes in depression samples, with activated dendritic cells, immature dendritic cells, monocytes, and neutrophils showing upregulation and activated B cells, activated CD4 T-cells, effector memory CD8 T-cells, and immature B cells showing downregulation (Figure 5A). These results suggested significantly altered immune cell profiles in MDD cases comparing with healthy controls (Figure 5B). BIRC3 exhibited the most obvious positive correlation with activated CD4 cells, with low scores in MDD (Figures 5C–E). Meanwhile, HMGB1 showed a significant negative correlation with monocyte. The score for HMGB1 was lower in MDD, while monocytes showed the opposite phenomenon (Figures 5F–H).

**FIGURE 6:**
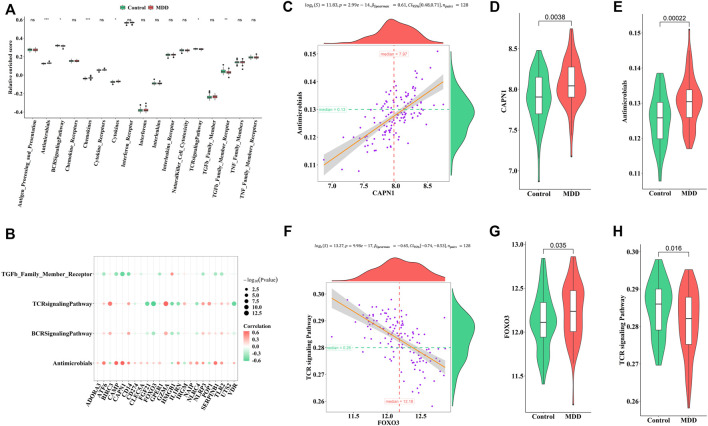
Correlation between pyroptosis-related genes and immune response geneset **(A)**. Differences in the abundance of immune response geneset between healthy control and MDD samples **(B)**. Correlation between pyroptosis-related genes and immune response geneset **(C–E)**. The most positive correlation between immune response geneset and pyroptosis-related genes **(F–H)**. The most negative correlation between immune response geneset and pyroptosis-related genes Adjusted *p*-values were showed as: ns, not significant; *p < 0.05; **p < 0.01; ***p < 0.001.

Similarly, the enrichment fractions of immune-related pathways were also calculated by ssGSEA (Figure 6A). We observed differences in the antimicrobial pathway, B cell receptor (BCR) signaling pathway, chemokines, cytokines, TCR signaling pathway, and transforming growth factor (TGF)-β family member receptors between healthy controls and MDD cases (Figure 6B). CAPN1 was positively correlated with antimicrobials (Figures 6C–E), while FOXO3 was negatively correlated with the TCR signaling pathway (Figures 6F–H). We speculated that these changes immune cells and immune-related pathways played important roles in occurrence and development of MDD.

### Identification of MDD Subtypes Based on 23 PS Genes

**FIGURE 7:**
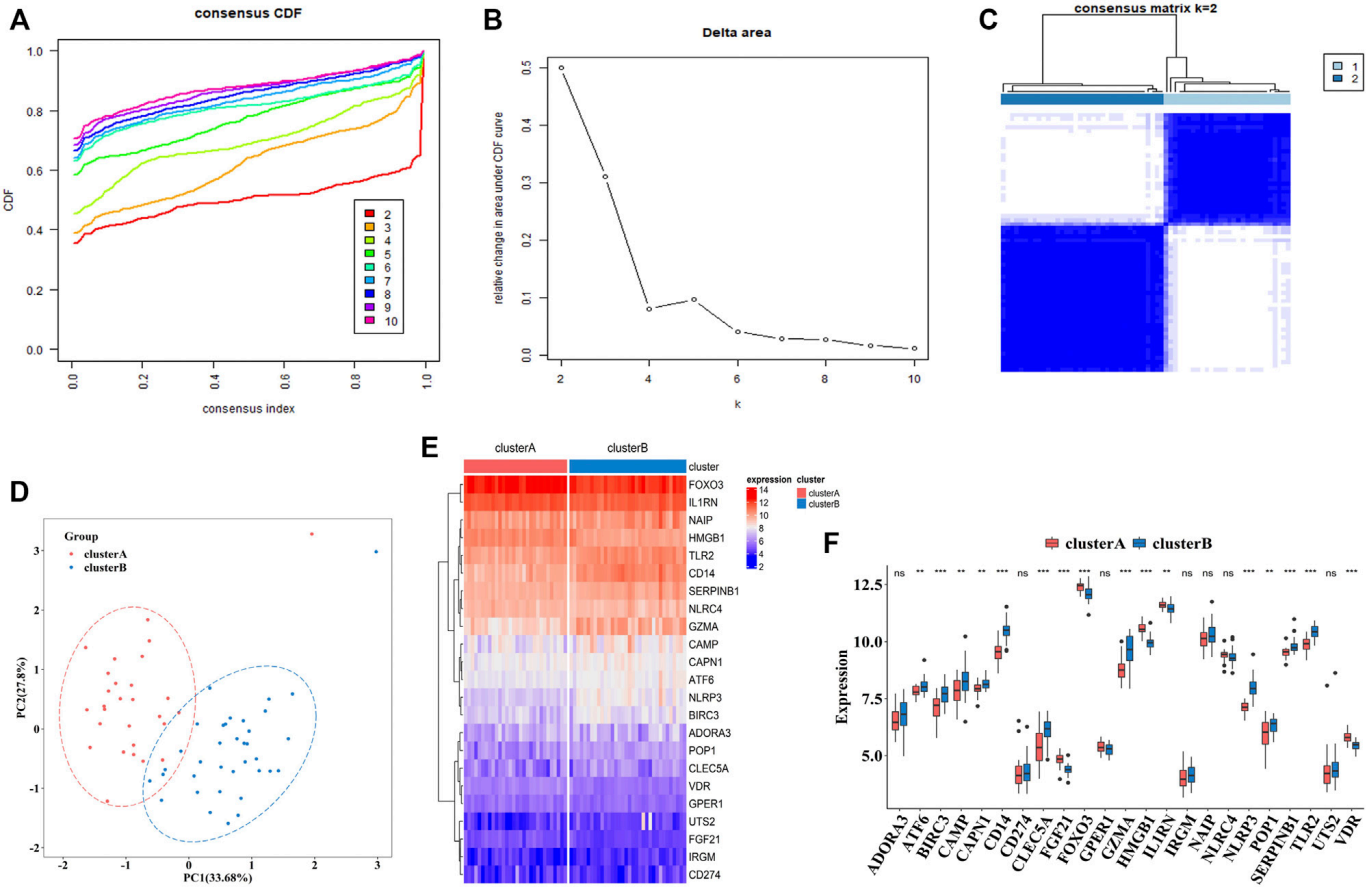
The construction of pyroptosis-related MDD subtypes **(A)**. The empirical cumulative distribution function (CDF) plots revealed the consensus distributions for each k (k = 2–10) **(B)**. Area change under CDF curve when K = 2–10. **(C)**. Two pyroptosis-related MDD clusters were generated *via* unsupervised consensus cluster **(D)**. PCA for the expression profiles of the 23 pyroptosis-related genes **(E,F)**. Expression heat map and boxplot map of the 23 pyroptosis-related genes in cluster A and cluster B. Adjusted *p*-values were showed as: ns, not significant; **p < 0.01; ***p < 0.001.

To explore the connections between the expression profiles of 23 PS genes (*CLEC5A, CAMP, TLR2, ADORA3, NLRP3, CD14, CD274, NLRC4, NAIP, CAPN1, GPER1, VDR, FGF21, IL1RN, FOX O 3, SERPINB1, ATF6, HMGB1, POP1, IRGM, BIRC3, GZMA,* and *UTS2*) and MDD subtypes, we performed unsupervised clustering using data from 64 MDD cases. By increasing the clustering variable (k) from 2 to 10, we observed the highest intra-group correlation and lowest inter-group correlation for k = 2, suggesting that the 64 MDD cases could be divided into two clusters according to these genes. Cluster A included 30 samples and cluster B contained 34 samples (Figures 7A–C).

PCA analysis indicated these two clusters differentiated significantly (Figure 7D). The heatmap and boxplot showed the expression differences of 23 PS genes between two clusters, in which the expressions of *ATF6, BIRC3, CAMP, CAPN1, CD14, CLEC5A, GZMA, NLRP3, POP1, SEPRINB1*, and *TLR2* were significantly increased in cluster B, while the expressions of *FGF21, FOX O 3, HMGB1, IL1RN,* and *VDR* were significantly decreased. *ADORA3, CD274, GPER1, IRGM, NAIP, NARC4,* and *UTS2* did not differ significantly (Figures 7E,F).

### Distinct Immune and Biological Characteristics Between Two Clusters

**FIGURE 8:**
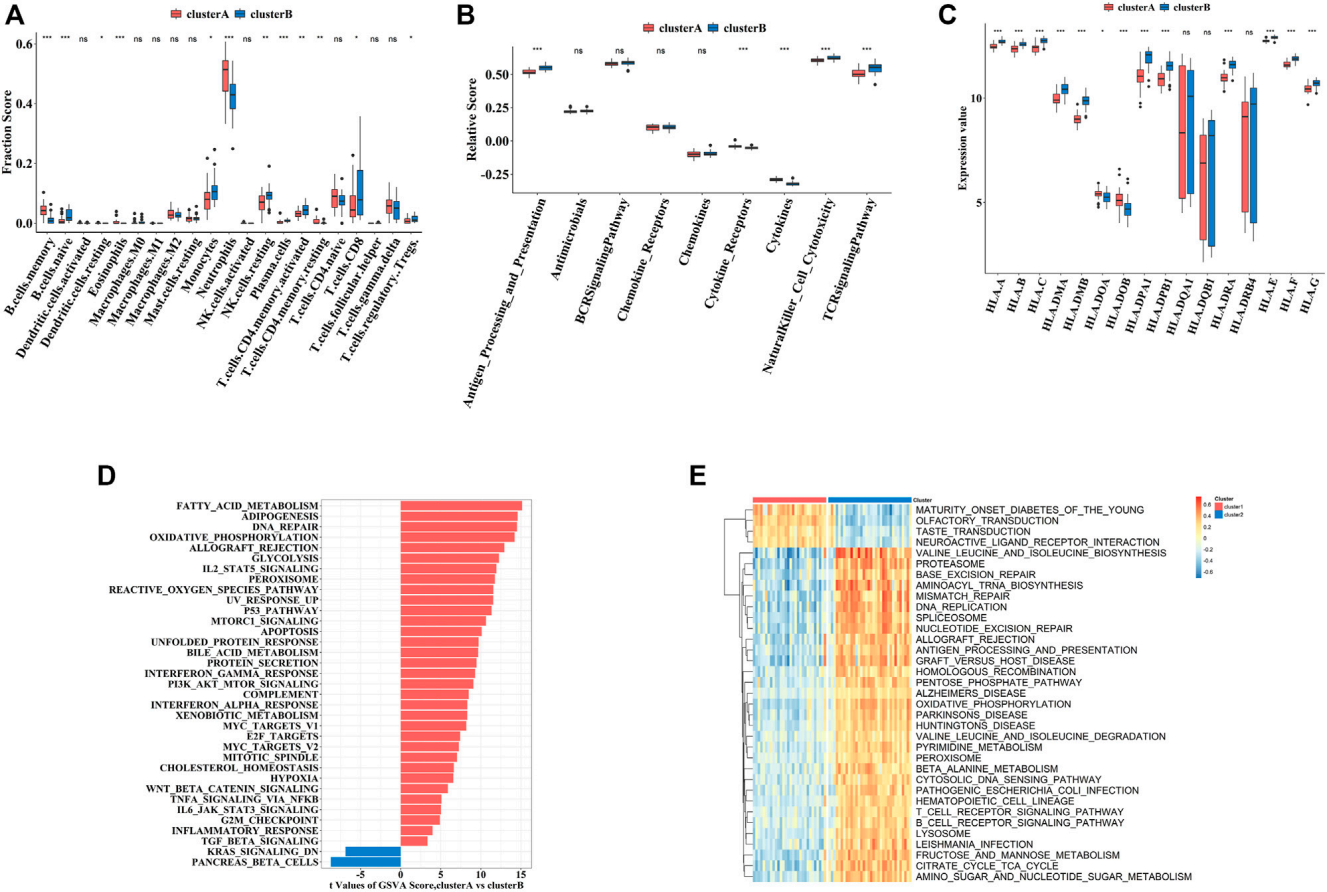
The immune and biological landscapes of two pyroptosis-related clusters **(A–C)**. The results of immune infiltrating cells **(A)**, immune response gene set **(B)**, and HLA gene set **(C)**: boxplot map of the enrichment scores among cluster A and cluster B **(D,E)**. The results of Hallmark pathways **(D)** and Kyoto Encyclopedia of Genes and Genomes (KEGG) pathways **(E)**: heat map of the enrichment scores among cluster A and cluster B Adjusted *p*-values were showed as: ns, not significant; *p < 0.05; **p < 0.01; ***p < 0.001.

To explore the immune characteristics between these two clusters, we measured the immune cells enrichment fractions, immune pathway activity, and human leukocyte antigen (HLA) gene expression profiles. The two clusters revealed completely different immune characteristics: for example, memory B cells and cytokines were mainly enriched in cluster A, while HLA genes mostly concentrated in cluster B (Figures 8A–C).

In addition to immune characteristics, we further explored the biological functions. We analyzed the enrichment scores of the Hallmark pathways and Kyoto Encyclopedia of Genes and Genomes (KEGG) pathways in the two clusters by GSVA, which revealed enrichment of pathways in cluster A, including fatty acid metabolism and neuroactive receptor interaction (Figures 8D,E).

### Generation of “PS-Scores” and Functional Annotation

**FIGURE 9:**
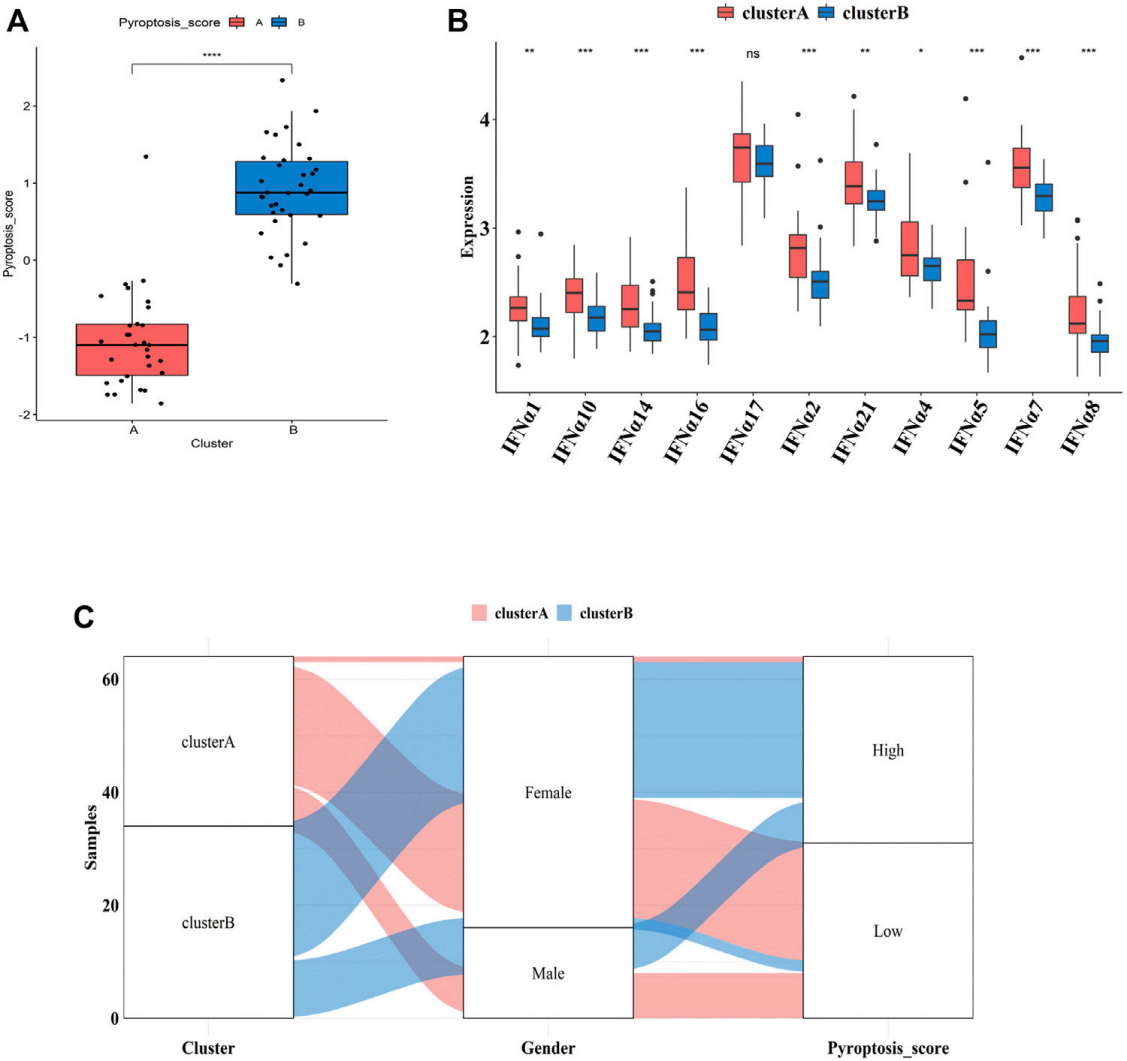
Differences in PS-scores and IFN-α isoforms between two pyroptosis-related clusters **(A)**. Differences in PS-scores between cluster A and cluster B **(B)**. The relationships between clusters, gender, and “PS-scores” **(C)**. Differential expression levels of IFN-α isoforms between cluster A and cluster B. Adjusted *p*-values were showed as: ns, not significant; *p < 0.05; **p < 0.01; ***p < 0.001.

To further explore the pathological mechanisms of MDD related to pyroptosis, we developed a pyroptosis-related signature score, the “PS-score,” including the phenotype-related genes to quantify the pyroptosis regulation pattern of each MDD sample. The “PS-scores” for two distinct subtypes were calculated. The “PS-scores” of cluster B was significantly higher than that of cluster A (Figure 9A). Figure 9B exhibited that the relationships between gender, pyroptosis clusters, and “PS-scores”.

IFN-α is currently suggested to be an important link in the pathogenesis of MDD. Clinical studies have shown significantly higher serum IFN levels in depression patients than those of normal people. After 12 weeks of antidepressant treatment, IFN levels were significantly lower than those before treatment (Shelton et al., 2011). Thus, we explored the relationship between two subtypes and expression profiles of IFN-α isoforms. Figure 9C indicated that the expression profiles of IFN-α1, IFN-α2, IFN-α4, IFN-α5, IFN-α7, IFN-α8, IFN-α10, IFN-α14, IFN-α16, IFN-α17, and IFN-α21 were all lower in cluster B, although the difference was not significant for IFN-α17.

## Discussion

Major depression is a debilitating mental illness and a leading cause of suicide (Ting et al., 2020). Pyroptosis is originally identified as a key mechanism in fighting infection, and a growing number of research suggests its role in the development of several diseases. However, the role of pyroptosis in MDD remains unclear. Our present study first constructed a diagnosis model for depression based on PS genes and then explored the role of pyroptosis in depression.

Currently, there is no clear boundary between normal and depressive behavioral manifestations (Wakefield et al., 2007); thus, the diagnosis of depression is subjective and difficult to implement (Pan et al., 2018). Previous studies have reported several diagnostic biomarkers for depression. Leday, the original author of the GSE98793 dataset, reported an AUC of 0.71 for 165 gene combinations (Leday et al., 2018). In addition, in two independent sample sets of patients with depression, Papakostas et al. (2013) reported high diagnostic performance and sensitivity and specificity >80% for nine biomarkers (alpha1 antitrypsin, apolipoprotein CIII, brain-derived neurotrophic factor, cortisol, epidermal growth factor, myeloperoxidase, prolactin, resistin, and soluble tumor necrosis factor-alpha receptor type II). We previously constructed a diagnostic model with the signature of four autophagy-related genes used GSE98793 dataset and autophagy gene set as well. The AUC of autophagy-related diagnostic model was 0.779 (He et al., 2021). In the present study, we explored a molecular diagnostic model for MDD based on PS genes and observed whether this model had higher efficiency. We firstly identified 23 differentially expressed PS genes between MDD cases and healthy controls. Further analysis using RF in machine learning identified *UTS2, NLRC4, GZMA, GPER1, IL1RN, CAPN1, NLRP3, HMGB1, ATF6*, and *ADORA3* as key PS genes affecting MDD classification. Then, the LASSO logistic regression finally screened feature genes and develop a diagnostic model based on eight genes (*GPER1, GZMA, HMGB1, IL1RN, NLRC4, NLRP3, UTS2, CAPN1*). Our present diagnostic model showed the AUC was 0.795 (95% CI: 0.721–0.868) for this model, indicating the high performance for differentiating MDD cases from healthy controls.

Of these eight genes (*GPER1, GZMA, HMGB1, IL1RN, NLRC4, NLRP3, UTS2, CAPN1*), *GPER1, IL1RN, NLRP3,* and *HMGB1* are reported associated with MDD previously. Pattern recognition receptors (PRRs) may play an important role in the interaction between inflammatory response and behavior. Several damage-associated molecular patterns (DAMPs) are associated with stress and depression, especially NLRP3 and HMGB1. The NLRP3 inflammasome is activated and GSDMD cleavage, subsequent IL-1β and IL-18 release, finally pytoptosis occurs. HMGB1 can induce NLRP3 activation, play an important role in pyroptosis as well.

The activation level of NLRP3 inflammasome was increased in multi-pathway induced depressed mouse models. Furthermore, the specific inflammasome inhibitor VX-765 blocked NLRP3 activation in the hippocampus and improved depression-like behavior in chronically unpredictable stressed mice. Clinical investigation reported increased NLRP3 mRNA and protein levels in peripheral blood mononuclear cells of MDD patients compared with healthy controls (Kaufmann et al., 2017). HMGB1 can be highly expressed in the cerebral cortex (Lian et al., 2017) and hippocampus (Liu et al., 2019) of CUMS mice. It is reported that stress can induce depression-like behaviours through the HMGB1/TLR4/NF-κB signalling pathway in the hippocampus (Liu et al., 2019) and PFC (Xu et al., 2020). G protein-coupled receptor 30 (*GPR30*), also known as G protein-coupled estrogen receptor 1 (*GPER1*), levels in MDD patients were significantly higher than that in the healthy controls (Findikli et al., 2017). GPER stimulates IL-1β secretion *via* JNK and p38 MAPK signaling pathways, and then induce pyroptosis (Deng et al., 2020). *IL1RN* (the gene encoding IL-1RA) encodes a classical signal peptide that secretes cytokines through the endoplasmic reticulum and Golgi apparatus (Lennard, 2017). The NLRP3 and caspase-1 affect the release of IL-1RA, which has a broad effect on the inflammatory response and pyroptosis in bladder epithelial cells (Lindblad et al., 2019). A meta-analysis reported significantly higher levels of cytokine receptor antagonists (IL-1RA) in MDD patients compared to those in normal controls (Goldsmith et al., 2016). However, no associations between the other six candidate genes and depression have been reported. We believe that our present results will provide a direction for future research on early diagnosis of depression based on pyroptosis.

A growing number of studies have reported the associations between pro-inflammatory cytokines and emotional, cognitive and behavioral changes, many of which are associated with depression. For example, higher levels of pro-inflammatory cytokines such as interleukin-6 and C-reactive protein have been discovered in MDD patients (Howren et al., 2009). Poor response to antidepressant treatment is obviously associated with elevated levels of proinflammatory cytokines (Hiles et al., 2012). Traditional antidepressants, such as fluoxetine and imipramine, had no obvious effects on more than 40% of MDD patients. Surprisingly, the use of anti-inflammatory drugs in conjunction with antipsychotics can relieve a range of psychotic symptoms (Berk et al., 2020). Therefore, we next observed the immune status of MDD patients and healthy controls in GSE98793 dataset. We found that activated dendritic cells, immature dendritic cells, monocytes, and neutrophils were higher, and activated B cells, activated CD4 T-cells, effector memory CD8 T-cells, and immature B cells were lower in MDD patients compared with healthy controls, with similar to Pfau’ (Pfau et al., 2018) and Kronfol’s studies (Kronfol, 2002). The up-regulation profiles of dendritic cells, monocytes, and neutrophils illustrate infection and inflammation occur and progress. Our results indicated MDD patients might present immune imbalance and inflammatory status.

Pyroptosis is a type of programmed cell death that is closely related to the inflammatory response. In the process of pyroptosis, cells form various vesicles, with pores 10–20 nm in diameter appearing on the cell membrane after gasdermin shear. It is reported that the release of many inflammatory factors leads to cascade amplification of cellular inflammatory responses (Frank and Vince, 2019), and pyroptosis is closely related to the immune response. We also discovered PS genes were closely associated with the changes of immune cells and immune-related pathway in GSE98793 dataset. Furthermore, our molecular diagnostic model based on pyroptosis showed a good performance. It was attractive for us to explore the immune-related mechanisms and search effective therapy based on pyroptosis can target a specific group of depression patients. So, we divided the MDD patients in GSE98793 into two subtypes according to pyroptosis, and found the immune characteristics and “PS scores” of subtypes were different. The “PS score” of cluster B was significantly higher than that of cluster A, and patients in cluster B presented lower profiles of B cells memory, dendritic cells, eosinophil, cytokines receptor pathway, and IFN-α family, which suggesting higher “PS score” might indicate lower levels of inflammatory condition. In addition, HLA genes play an important role in immune response and immune therapy. Attractively, most HLA genes were up-regulated in cluster B, indicating that patients in cluster B might be more sensitive to immune therapy. Previous studies identified different pyroptosis-related subtypes for gastric cancer and melanoma, providing a new method for the prognosis and survival of tumor patients and promoting the development of personalized therapy (Liu et al., 2021). According to our results, deep exploration of immune regulation in depression could help us understand and develop accurate and effective anti-inflammatory therapy for MDD patients. However, unfortunately, the clinical information of MDD patients is very limited, so we only conducted a partial study to observe the phenomenon, and further study is needed.

Few studies have assessed the role of pyroptosis in depression. This preliminary study explored the diagnostic values of PS genes in depression, and systematically analyzed the relationships between PS genes and the immune response in depression, providing theoretical support for future research. However, our study has some limitations. First, the total sample size was relatively small (MDD: *n* = 64; Normal: *n* = 64). Secondly, this study was based on bioinformatics analysis; thus many of the results were theoretical. However, as experiments are the only standard for verifying the results, their accuracy requires verification without experimental methods. Third, due to the lack of abundant clinical data, we cannot determine the specific role of these PS genes in depression, which warrants further study.

In summary, our study provided a molecular model for MDD diagnosis. We additionally revealed the pyroptosis was closely related with immune imbalance in MDD. Comprehensive analysis of the pyroptosis pattern of MDD will improve our understanding of the internal mechanism of the MDD immune regulatory network and inform the development of more effective treatment methods.

## Data Availability

The datasets presented in this study can be found in online repositories. The names of the repository/repositories and accession number(s) can be found in the article
